# Context‐Appropriate Clinical Practice Guidelines for the Care of Explosive Ordnance Casualties in Low‐Resource Settings

**DOI:** 10.1002/wjs.12644

**Published:** 2025-05-30

**Authors:** Nina Weston, Aparna Cheran, Amila Ratnayake, Adam L. Kushner, Hannah Wild

**Affiliations:** ^1^ Medical School University of Cambridge Cambridge UK; ^2^ Explosive Weapons Trauma Care Collective International Blast Injury Research Network Southampton UK; ^3^ Military Hospital Narahenpita Colombo Sri Lanka; ^4^ Surgeons OverSeas New York New York USA; ^5^ Department of Surgery University of Washington Seattle Washington USA

**Keywords:** clinical practice guidelines, explosive ordnance injuries, low-resource settings

## 1 Introduction

Between 2022 and 2024, civilian fatalities from explosive weapons (EW) increased by 122%, attributable to the extensive use of EW in conflicts globally, including Ukraine, Gaza, Syria, Sudan, and others less publicized such as the Sahel [1, 2]. EW are indiscriminate by nature. In conflicts characterized by their use, the majority of casualties are civilian. The protracted nature of modern armed conflict, increasing complexity in involvement of nonstate armed groups, and disregard for international humanitarian law in combination with the use of EW in populated areas severely strains local healthcare infrastructure, limiting the quality of timely trauma care for local casualties [3]. Illustrating this disparity, the mortality rate among civilian victims of EW is approximately 38%, which is several times higher than that observed in high‐resource trauma systems [4].

Trauma care advances in high‐resource systems (military or civilian) have not yet systematically reached care practices in low‐resource conflict settings (LRCS). Among these advances are Clinical Practice Guidelines (CPGs), which have been described as the “backbone” of the Joint Trauma System (JTS) performance improvement program [5]. The JTS, under the US Department of Defense (DoD), aims to reduce variability in trauma care practice through developing and implementing CPGs system‐wide, with topics ranging from antibiotics to drowning management. Targeted toward deployed military medical professionals caring for injured service members, CPGs have since often been used in civilian care; the DoD itself recognized that “standards and recommendations have found their way into the civilian medical communities” [5].

Depending on medical rules of engagement at a given military treatment facility (MTF), civilian casualties may receive care at high‐resource MTFs within the JTS [6]. However, most local casualties receive care locally, for example, in public health facilities. JTS CPGs may not be optimally suited for local casualty care in these lower‐resourced environments. Potential issues include:

1. The CPG development process and author teams being exclusively from high‐resource militaries
2. The demographic differences between civilian and military casualties
3. Differences in resources available to humanitarian versus military healthcare professionals, including limited evacuation abilities

This editorial perspective elucidates these differences, illustrating the need for context‐appropriate CPGs, and provide an overview of a potential solution: new CPGs being developed for local casualty care by the Explosive Weapons Trauma Care Collective (EXTRACCT); the development process details are included here as Online Resource 1. EXTRACCT is a multisectoral initiative to reduce preventable death and disability among EW victims in LRCS [7]. Widespread use of CPGs designed specifically for local casualties in LRCS holds potential to improve the quality of care provided to EW victims.

## 2 CPG Development Process

The development process and objectives of JTS versus EXTRACCT CPGs vary slightly. The former are based on DoD Trauma Registry data, after action reports and predominantly military patient records. JTS CPGs, designed for implementation in well‐resourced MTF by military medical professionals of various clinical backgrounds, aim to “remove medical practice variations and save lives” [5]. The EXTRACCT CPGs have similar goals, but aim additionally for embedded minimum casualty data requirements for “longitudinal monitoring of patient outcomes”, given the lack of system‐wide local casualty registries in LRCS. In addition to content derived from JTS CPGs, EXTRACCT CPGs incorporate evidence synthesized from the literature on trauma management in LRCS, and expert opinion where this evidence base does not exist. Although JTS CPGs are developed by subject matter experts from high‐resource settings, EXTRACCT CPG development features equal or greater representation from trauma care providers in LRCS to ensure applicability and appropriateness to the target context [7, 8]. Over time, continued refinement of the EXTRACCT CPGs based on local casualty data will improve their specificity for LRCS where treatment algorithms may differ from those best‐suited to high‐resource military trauma systems. Additionally, EXTRACCT CPGs increase their accessibility to a variety of practitioner levels through simple language and emphasis on visual formatting, as well as concise and highly practical content. Such adaptations are necessary as LRCS health professionals are more likely to be generalists in comparison to their counterparts in higher‐resource settings, who receive more standardized training specialized to conflict zones.

## 3 Demographic Differences Between Civilian and Military Casualties

DoD CPGs are not designed to consider differences of demography in civilian casualties. In 2023, the proportion of women and children in conflict doubled and tripled, respectively, from the year prior [9]. Pregnant women, children, and the elderly have unique clinical needs requiring targeted guidance absent from dedicated DoD CPGs. Despite significant overlap between topics covered by DOD and the projected EXTRACCT CPGs (e.g., vascular injury and burns), certain demography‐specific topic areas such as blast trauma in pregnant patients and pediatric blast injury will be a unique characteristic of the EXTRACCT CPGs [9].

## 4 Differences in Resources Available to Military Versus LRCS Healthcare Providers

One of the most significant differences between treatment in military trauma systems versus local healthcare systems in LRCS is the potential disparity in available resources. Although JTS guidance on prolonged field care resembles some of the austere conditions faced routinely in LRCS health systems, possibilities are limited for evacuation “out‐of‐theater” or to higher echelons of care for local casualties. With such limitations in mind, EXTRACCT CPGs often recommend “minimum” courses of treatment requiring little to no medical equipment, or that can be improvized with locally available materials. They also contain practical guidance on compounding of certain medications that are challenging to access in LRCS, such as the manufacture of antibiotic eye ointment from parenteral antibiotics in the forthcoming CPG on managing ocular blast injury.

Reflecting the unfortunate limitations in human and material resources within local healthcare systems in LRCS, EXTRACCT CPGs are realistic in their prognoses for injury patterns associated with high mortality rates. There is stronger emphasis on, and an entire set of forthcoming guidelines dedicated to, palliative care approaches. For example, burn injuries are one of the only triage criteria that differ between host nation and military service member casualties at DoD military treatment facilities. As stated in the DoD Burn Care CPG: “Triage of local national casualties to an expectant category may be required if their burns exceed the host nation's ability to treat and rehabilitate (e.g., full thickness burns > 50% TBSA)” [10]. The forthcoming EXTRACCT Burns CPG provides guidance consistent with these principles, and palliative considerations will be emphasized in a context‐appropriate manner throughout the EXTRACCT CPGs.

## 5 Conclusion

The JTS CPGs set the standard for combat casualty care within military trauma systems, but do not fully encompass the breadth of care requirements for local casualties of explosive injury in LRCS. Challenges in attaining best‐practice standards in LRCS harm *both* patients, and the mental wellbeing of healthcare workers. The forthcoming EXTRACCT CPGs address these gaps, firstly, through all CPGs being co‐developed by healthcare professionals working within LRCS. Secondly, EXTRACCT CPGs will include context‐appropriate “minimum” guidance more suitable for LRCS. Finally, the broader content domains encompassed by the EXTRACCT CPG initiative (e.g., special considerations for transport, pediatric, and palliative care) are absent from JTS guidelines [7]. This is understandable as the JTS CPGs, targeted toward the personnel and equipment available within well‐resourced military trauma systems, would not frequently require these considerations. Overall, the EXTRACCT CPGs have the potential to contribute significantly to clinical performance improvement for local casualties of explosive injury in LRCS.

## Author Contributions

**Nina Weston:** writing – original draft. **Aparna Cheran:** writing – review and editing. **Amila Ratnayake:** writing – review and editing. **Adam L. Kushner:** writing – review and editing, conceptualization. **Hannah Wild:** writing – review and editing, project administration, conceptualization, supervision.

## Conflicts of Interest

The authors declare no conflicts of interest.

## Data Availability

Data sharing not applicable to this article as no datasets were generated or analyzed during the current study.
